# Supplementary figures and images for: A viral vaccine design harnessing prior BCG immunization confers protection against Ebola virus
Source: Front Immunol. 2024 Jul 16;15:1429909. doi: 10.3389/fimmu.2024.1429909 (PMC11286471; doi:10.3389/fimmu.2024.1429909)

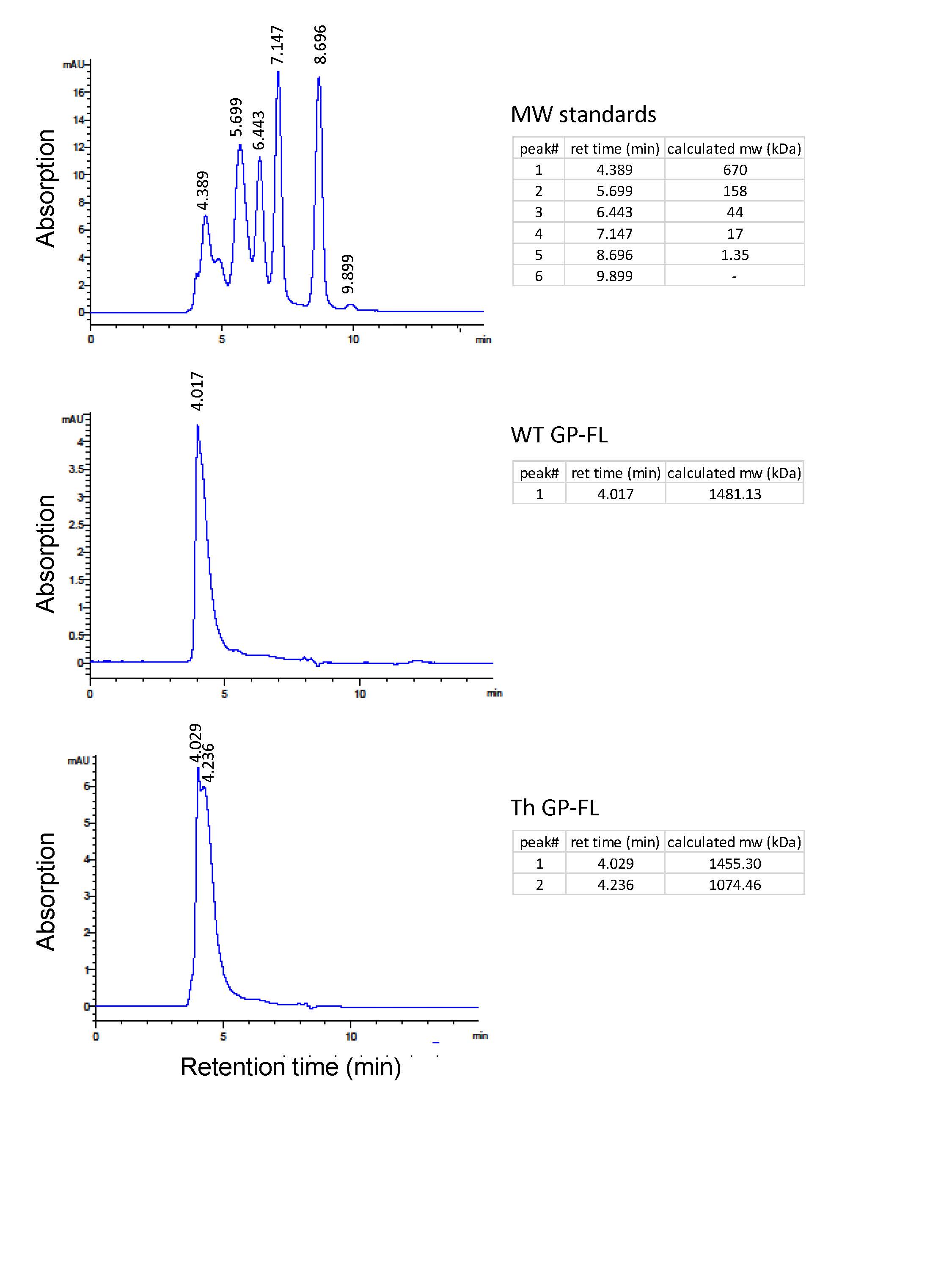

Supplement: Supplementary Figure 1 — Analysis of EBOV GP by size exclusion chromatography. Purified wildtype EBOV GP full-length (WT GP-FL) and the Th EBOV GP vaccine (Th GP-FL) were subjected to the SRT SEC-300 size exclusion column. SRT SEC-300 exclusion limit is 1250 kDa. [file Image_1.jpeg]

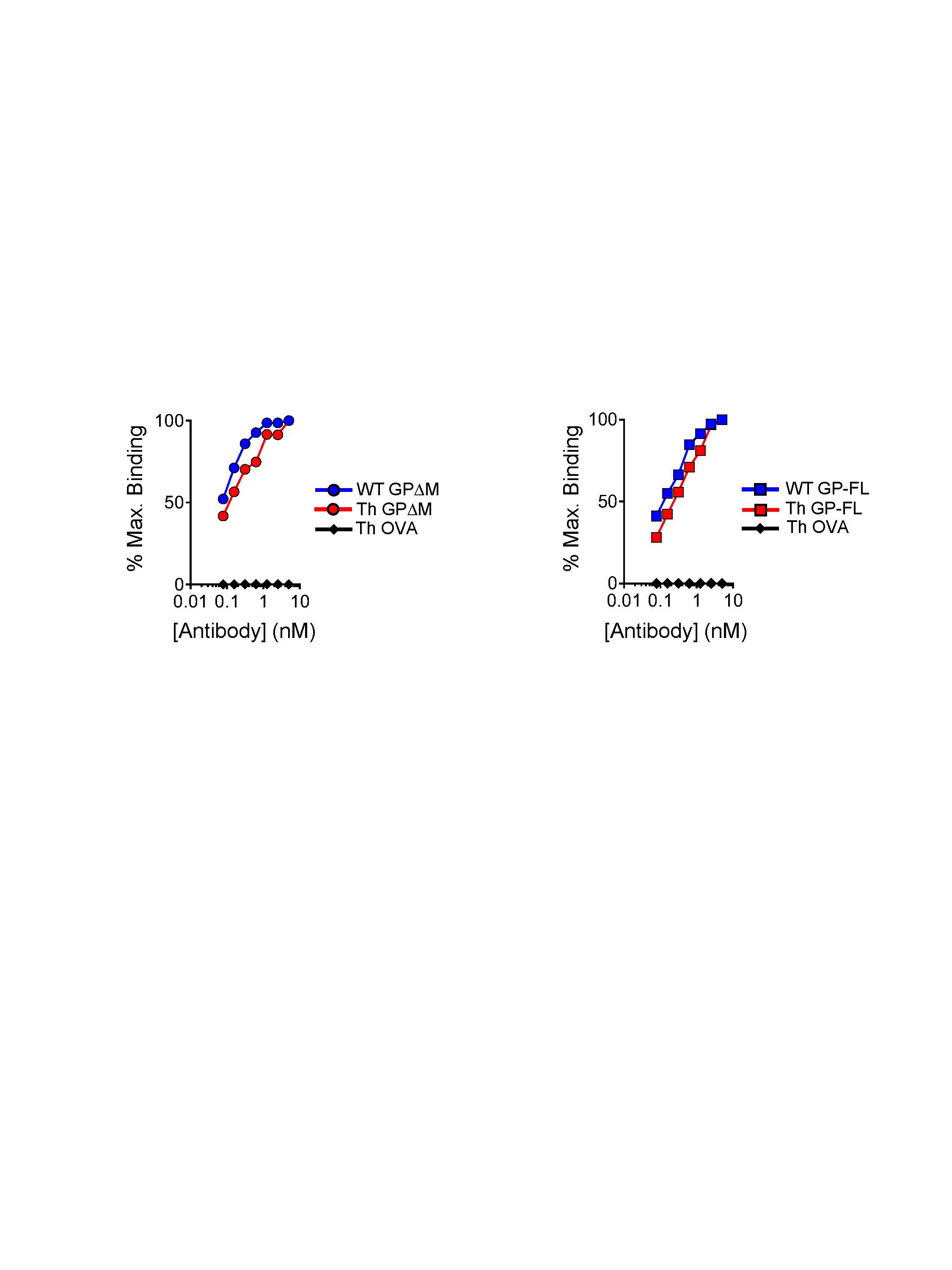

Supplement: Supplementary Figure 2 — Comparison of EBOV GP vaccines. (A) ELISA with antibody KZ52 specific for EBOV GP conformational epitopes was used to probe purified EBOV GP. The ovalbumin version of the Th vaccine (Th OVA) served as a negative control to show the specificity of KZ52 antibody against EBOV GP (left panel). [file Image_2.jpeg]

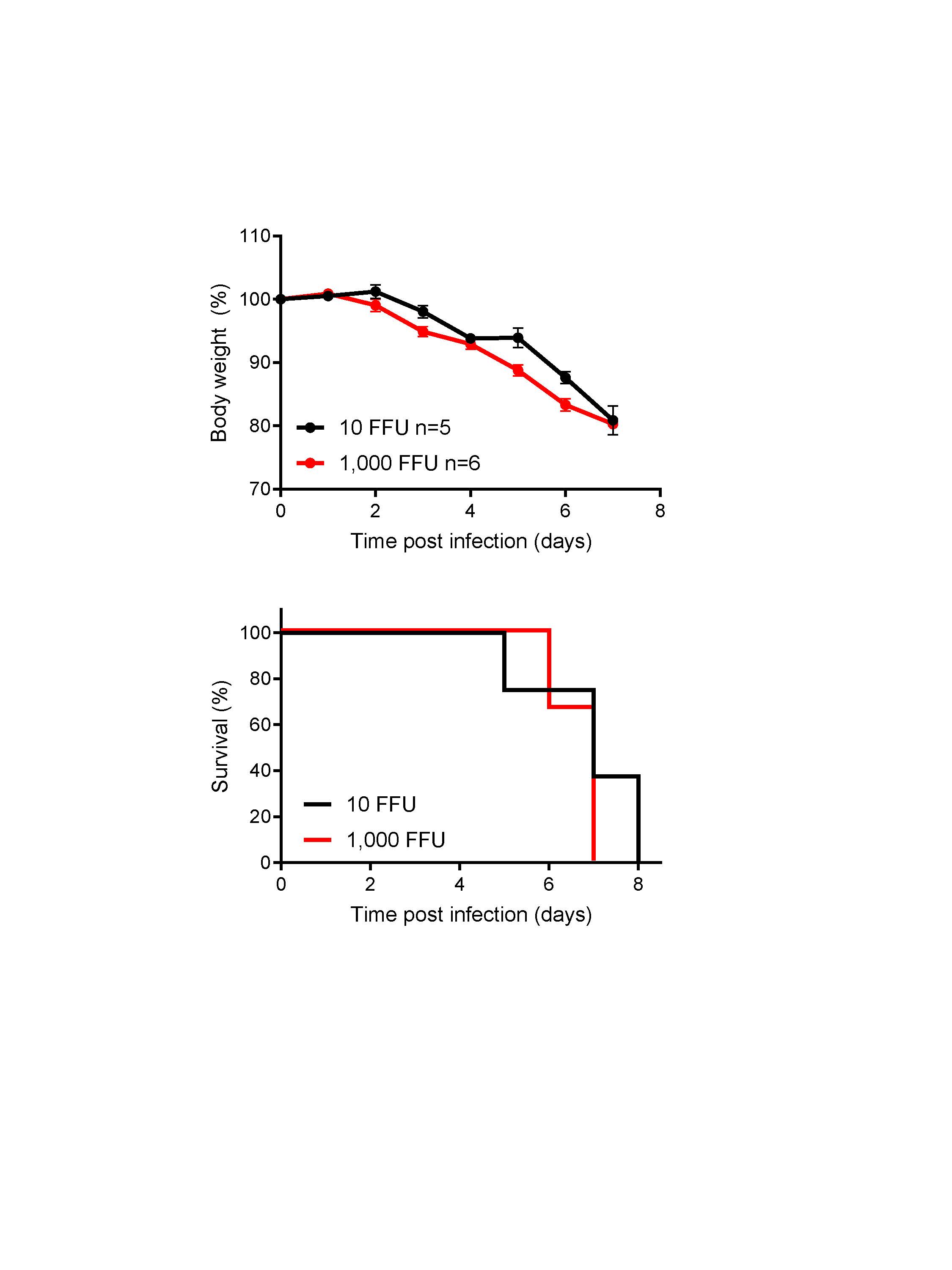

Supplement: Supplementary Figure 3 — Susceptibility of 24-week old C57BL/6N to MA-EBOV challenge. 24-week old mice were infected with 10 or 1000 FFU of mouse-adapted (MA-) EBOV and succumbed to infection by day 7-8 after EBOV challenge. [file Image_3.jpeg]
